# Electrochemical and Optical Biosensors for the Detection of *Campylobacter* and *Listeria*: An Update Look

**DOI:** 10.3390/mi10080500

**Published:** 2019-07-27

**Authors:** Priya Vizzini, Matteo Braidot, Jasmina Vidic, Marisa Manzano

**Affiliations:** 1 Department of Agriculture Food Environmental and Animal Sciences, University of Udine, 33100 Udine, Italy; 2 Micalis Institute, INRA, AgroParisTech, Université Paris-Saclay, 78352 Jouy-en-Josas, France

**Keywords:** food pathogens, *Listeria monocytogenes*, *Campylobacter*, electrochemical biosensors, optical biosensors

## Abstract

Foodborne safety has aroused tremendous research interest in recent years because of a global public health problem. The rapid and precise detection of foodborne pathogens can reduce significantly infection diseases and save lives by the early initiation of an effective treatment. This review highlights current advances in the development of biosensors for detection of *Campylobacter* spp. and *Listeria monocytogenes* that are the most common causes of zoonosis. The consumption of pathogen contaminated food is responsible for humans hospitalization and death. The attention focused on the recognition elements such as antibodies (Ab), DNA probes and aptamers able to recognize cells, amplicons, and specific genes from different samples like bacteria, food, environment and clinical samples. Moreover, the review focused on two main signal-transducing mechanisms, i.e., electrochemical, measuring an amperometric, potentiometric and impedimetric signal; and optical, measuring a light signal by OLED (Organic Light Emitting Diode), SPR (Surface Plasmon Resonance), and Optical fiber. We expect that high-performance of devices being developed through basic research will find extensive applications in environmental monitoring, biomedical diagnostics, and food safety.

## 1. Introduction

Bacterial contamination of food is a central issue of food safety because of the high incidence of foodborne diseases [1,2]. The European Food Safety Authority (EFSA) and the European Centre for Disease Prevention and Control (ECDC) have reported about 359,700 hospitalizations due to confirmed zoonoses with 500 fatal cases in EU in 2016 [3,4]. The majority of infections is caused by 15 pathogenic bacteria including *Salmonella*, *Campylobacter*, *Listeria* and Shiga toxin producing *Escherichia coli* *(STEC).* The global incidence of foodborne disease is difficult to estimate because many cases are not declared (especially minor outbreaks) or misdiagnosed. In addition, used tests cannot be performed on the point-of-need but samples have to be collected and transported in specialized laboratories, practice that contributes to underestimate the number of foodborne infections, and thus to their inefficient monitoring and control. Rapid and sensitive bacterial detection is a key element for efficient prevention of foodborne diseases [5].

Food industries need new analytical tools to monitor food production to avoid bacterial contamination, which leads to food recalls and consequent economic losses. Moreover, advanced detection methods are needed to meet the strict and specific regulatory guidelines on food security [6]. To respond to the food industry demands, the detection techniques should be user-friendly and autonomous, allowing analysis on-point-of-need without any specialized sample processing.

Identification and quantification of bacteria mostly rely on conventional, culture-based methods, and bacterial phenotypical characterization. Such traditional methods for pathogen detection are however inadequate to be performed in closed, confined spaces, as production plants. The culturing of pathogen microorganisms requires an enrichment step in a broth followed by bacterial growth on agar plates, and biochemical and physiological tests for strain identification. Such procedures are labor-intensive and time-consuming. In fact, it takes from three to five days to provide initial results, and in some cases up to few weeks for confirming the specific pathogenic strain. Alternative molecular methods based on PCR are faster, but need isolated genetic materials, specific instrumentation and trained personnel to be performed, and they are not suitable for rapid and point-of-care analysis.

Biosensor technology has attracted increased interest for a variety of applications in healthcare, agriculture and environmental monitoring. Biosensors offer the specificity and sensitivity of biological systems integrated into small, low-cost devices. Thanks to their high analytical performance, biosensors represent a powerful alternative to conventional methods in pathogen detection. Biosensors may integrate specific nucleic acid sequences, proteins (like antibodies or enzymes), and even whole cells or cell elements as recognition elements for a specific pathogen. The sensitive detection is enabled due to the transduction of molecular recognition of the target pathogen to a measurable signal that is processed and displayed.

Here, we present major recent advances in the development of electrochemical and optical biosensors to detect *Campylobacter* spp. and *Listeria monocytogenes*, foodborne pathogens that represent an important global health threat. Campylobacteriosis is one of the most frequent zoonoses notified in the EU, while listeriosis has the highest hospitalization and mortality rate among zoonotic diseases reported in the EU [3,6].

## 2. *Campylobacter* spp. and *Listeria monocytogenes*

*Campylobacter* is a Gram negative bacterium belonging to the family of *Campylobacteraceae*, with a spiral, rod or curved shape, and a small size, from 0.2 to 0.8 μm by 0.5 to 5 μm. The bacterium is oxidase and catalase positive. The human pathogenic species (*C. jejuni*, *C. coli*, *C. lari* and *C. upsaliensis*) are thermotolerant, with the optimum grown temperature between 37–42 °C. The presence of single polar or bipolar flagella, depends on the species. These microorganisms are able to move with corkscrew movement, important for their pathogenicity [7]. *Campylobacter* is considered a fastidious microorganism since it requires special culture conditions such as the addition of blood to a culture medium, and obligate microaerophilic conditions (2% H_2_, 5% CO_2_, 5% O_2_, and 88% N_2_) to grow [8]. Thus, the detection of *Campylobacter* can be difficult using both traditional culture medium and innovative methods like biosensors [5,9,10,11]. Furthermore, other aspects can influence negatively the detection. The bacteria can enter in a viable but not culturable (VBNC) status under stressing conditions. The detection based on culturing is not possible because they do not grow. The identification of *Campylobacter* by the optical microscope is not easy neither as bacterial cells lose their spiral distinctive shape, they can change their shape into spherical or coccoid [12]. Moreover, the current problem, revealed from Habib et al. [13], concerns the utilization of a not enough selective medium. The difficulty to detect *Campylobacter* in food matrices [14], entailed a revision of official ISO 10272 method [15] as a consequence of the resistance developed against the antibiotic added to the enrichment broth from the extended spectrum beta-lactamases (ESBL) bacteria. The main sources of *Campylobacter* for human contamination are broiler [16], undercooked chicken [17] and in minor way milk, fruit vegetables and water [18].

*Listeria monocytogenes,* a Gram positive bacterium, rod shaped with a size of 0.4 to 0.5 μm by 0.5 to 2 μm, is ubiquitous and belongs to the family of *Listeriaceae*. The bacterium is oxidase negative and catalase positive, and can grow on simple culture media with an optimum temperature at 37°C. It can survive at pH from 4.3 to 9.6, and at 20% of NaCl [19]. *L. monocytogenes* owns flagella that allow for the invasion of the host.

It is able to form biofilms on several surfaces such as clinical catheters and food sources [20]. Moreover, there is an emergency problem of the antibiotic resistance of *Listeria* [21,22].

The main sources of human contamination with *L. monocytogenes* are Ready to Eat (RTE) foods [23], followed by raw milk and dairy products [24], and seafood products [25].

To reduce incidence of human infections by *Campylobacter* spp. and *Listeria*, it is important to find specific, sensitive and suitable analysis methods to deal with the current detection problems of both pathogens.

## 3. Detection of *Campylobacter* spp. and *Listeria monocytogenes* by Electrochemical Biosensors

### 3.1. Electrochemical Biosensors

An electrochemical biosensor can be ion selective, glass, metal and carbon electrode. The electrochemical signal is generated either by the presence of the analyte on the electrode surface or a direct formation of electroactive species by the target molecule (analyte) or indirectly by coupling a biorecognition event with a redox probe or a mediated enzyme electrode [26]. The measurement modes are amperometry, potentiometry, conductometry and impedimetry. The amperometry is based on the current signal resulting from the electrochemical oxidation or reduction of electro-active species. The amperometric signal due to the application of the current depends on the electro-active species oxidation reaction at electrode level. The increase or decrease of resistance causes a variation in the current signal.

The potentiometry measures the potential difference between a working and reference electrode at equilibrium. The conductometry monitors the changes in the electrical conductivity of the sample solution taking into account the resistance caused by the components present in the solution. Electrochemical impedance spectroscopy (EIS) is a method of transducing analyte interaction on electrode surfaces under specific system parameters. Thanks to its high sensitivity EIS is a rapidly developing electrochemical technique for the transduction of biosensing events on electrodes. The use of the electrochemical impedance spectroscopy (EIS) is becoming more common and can determine both the resistive and capacitive (dielectric) properties of materials in the biosensor. EIS methods were often used for bacterial identification and quantification by monitoring the changes in the impedance of the solutions, induced by release of ionic metabolites from living cells (i.e., carbon dioxide, and organic acids). Compared to other electrochemical techniques, EIS treats the dynamics of an electrochemical process by linearization of current−potential characteristics [27,28]. For all of these electrochemical detections the change of signal is proportional to the concentration of the analyte. One of the most important elements of an electrochemical sensor is the material of the working electrode. Usually, inert metals, like gold or silver, or carbon-based materials are used as they are chemically resistant to biological materials. In recent years, electrodes are frequently modified with a range of nanoparticles or conducting polymers to increase their analytical characteristics, such as improved electrical conductivity, specificity or increased electrode surface area. Moreover, to improve the detection signal conducting polymers can be used, such as copolypyrrole integrated with ferrocenyl group as redox marker [29]. Another example is the use of silver nanoparticles (AgNPs) coated with a mixture of zwitterionic and biotinylated zwitterionic polymers [30]. Thanks to these expedients the electrochemical oxidation strongly enhances the detection signal.

To enable detection, the recognition element, called bioreceptor, is immobilized on the electrode in a stable manner, usually by covalent binding. Bioreceptors have a high affinity toward a target analyte (bacterial biomarker). Recognition elements can be an antibody if a biomarker is a specific bacterial protein or a DNA probe when a specific nucleic acid sequence is targeted, or aptamers when the target is a cell. The immobilization of the bioreceptors can be conducted by several methods, the mains are: adsorption, covalent bonding and crosslinking. The adsorption is a physical method which consists in adhesion of atoms, ions, biomolecules to a surface, while the covalent bonding and crosslinking are chemical methods. A DNA probe or an aptamer with a thiol tag at 5′ or 3‘end, can covalently bind gold electrodes. Crosslinking usually is obtained by a glutaraldehyde treatment of the electrode surface to motivate the bioreceptor covalent linkages. An alternative is a surface self-assembled monolayer (SAM) [31,32] which could contain OH, –NH_2_, –COOH, or –SH groups. 

### 3.2. Electrochemical Detection of Campylobacter spp.

Detection of *Campylobacter* spp. by electrochemical biosensors was reported in few studies during the last 20 years. The first label-free electrochemical biosensor based on amperometry to detect *Campylobacter* was described by Ivnitski et al. [33]. The biosensor was based on a lipid bilayer membrane (BLM), deposited on stainless-steel working electrode used as a transducer. The BLM acted as an electric insulator layer on the electrode. The antibodies embedded into BLM, used as bio-receptors, were able to bind bacterial cells, which changed the permeability of the layer and the current intensity and thus allowed detection. Indeed, the binding between the specific antibody and *Campylobacter* cell induces an increase of ions flux through channels opened in the membrane and a consequent current pulse. The detection was highly sensitive and specific, and provided results within 10 min. No blocking solution was needed to eliminate background signal or enrichment step to increase the number of bacterial cells. The limit of detection (LOD) of 1 cell was reported considering the ions-flowing concentration that goes through the channels. Although this approach is promising in flow mode application, it requires an additional step to concentrate cells for application in food analysis.

Che et al. [34] studied a method to detect *Campylobacter jejuni* in water used to wash inoculated chicken carcass and ground turkey meat using immunomagnetic separation (IMS). Magnetic beads of several types and sizes, and blocking solutions were tested using antibodies for *C. jejuni*. Beads coated with antibodies anti *C. jejuni* were added to a sample solution, and after, a solution of phosphatase labeled antibodies was added. The mixture was then subjected to IMS separation before the electrochemical detection using the electrochemical carbon paste biosensor coupled with an enzymatic reaction (95 mg carbon paste +5 mg tyrosinanse). Cells trapped on the magnetic beads produced phenol in the presence of TBS (Tris Buffered saline), MgCl_2_ and phenil phosphate.

The LOD obtained was of 2.1 × 10^4^ CFU·mL^−1^. The overall time needed for the analysis, including separation and detection, was 3.5 h.

The new generation of screen-printed electrodes provides portable and disposable electrochemical biosensors. Viswanathan et al. [35] developed a screen-printed electrode composed of carbon-nanotube carrying specific antibodies. The aim was the simultaneous detection of several pathogens such as *Campylobacter, E. coli* and *Salmonella* in spiked milk. After bacterial capturing by the immobilized antibodies, the detection signal was obtained using a second antibody labelled with nanocrystals (PbS, CuS and CdS) which releases metal ions easily detectable by square wave anodic stripping voltammetry. The dynamic range from 1 × 10^3^ to 5 × 10^5^ cell·mL^−1^ was observed. The detection limit for all tested pathogens was the same, 400 cell·mL^−1^. Che et al. [34], and Viswanathan et al. [35], did not use a blocking solution to avoid unspecific signals.

**Figure 1:**
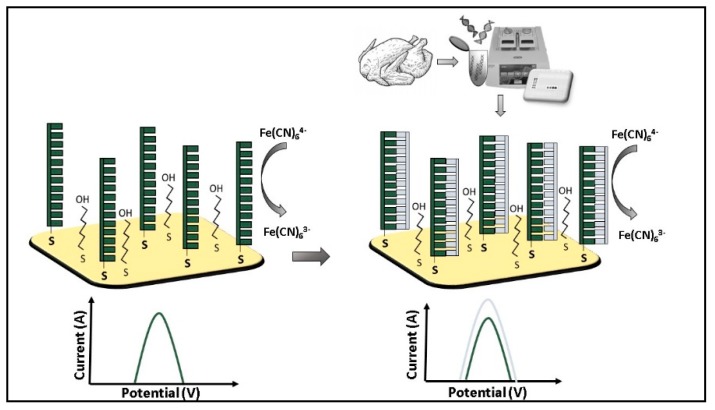
*Campylobacter* spp. amplicons detection by electrochemical biosensor based on differential pulse voltammetry (DPV) using Fe(CN) as an indicator [36].

Morant Minana and Elizalde [36] developed a DNA electrochemical biosensor (Figure 1) to detect *Campylobacter* using as a target amplicon of 181 bp of the gene *flaA,* which encodes for a flagellin protein that polymerizes to form the filaments of the bacterial flagella, and is present only in *Campylobacter* spp.

The working electrode was an Au-microscale electrode with Cyclo Olefin Polymer [37] functionalized with the DNA probe able to hybridize amplicons of the gene *flaA*. The probe was labeled at 5′ with a thiol group to allow it immobilization on the Au electrode. The unspecific signal was avoided using 6-mercaptohexanol (MCH) as a blocking agent mixed with the DNA probe, to give higher irreversibility of the functionalization. Ferricyanide [Fe(CN_6_)^3−^] was used as a redox probe in voltammetry measurements. The signal decreased after each immobilization step and after the probe hybridization with the target. The decrease in current intensities was explained by a decrease of the electron transfer from the [Fe(CN_6_)^4−^] to the gold surface due to the electrostatic repulsion induced by the presence of the DNA probe and the hybridized sequence. This is caused by the negative charge of the phosphate groups present on the external DNA scaffold [38]. Indeed, an anodic peak decrement shown in comparison with bare gold electrode and the subsequently immobilized DNA probe, was explained as a signal decrease due to the presence of the hybridized DNA target. This trend was due to the overlapping of the atomic orbitals along the DNA chain that prevent ferricyanide oxidation and reduction on the electrode [39]. When DNA target amplicons of 181 pb and genomic DNA were tested a LOD of 9 × 10^−11^ mol·L^−1^ was obtained.

Moore et al. [9], Falahee et al. [10] and Line et al. [11] studied the detection of *Campylobacter* grown in several broths using impedimetric or conductimetric systems. The proliferation of the bacteria in a growth medium changed the conductivity. However, the experimental approach was not optimized, as not-specific impedance changes could have been produced from bacterial contaminants present in the growth medium. Recent data confirmed the inadequacy of the existing broths [40]. However, the selective medium cannot solve the problem caused by the VBNC status, which reduces the detection capability, and increases the time required for analysis.

To the best of our knowledge, only one immunosensor based on impedimetry was developed to detect *Campylobacter*. Huang et al. [41] applied this sensor to test stool samples after a step-in enrichment broth to multiply bacterial cells. The sensor was based on O-carboxymetilchitosan electrode modified by Fe_3_O_4_ nanoparticles (NPs) on which the anti-flagellin antibody was immobilized. The main advantages of this approach were the increased electrode active surface used for immobilization obtained due to NPs, the possibility to detect label-free flagellin, and the easy to perform regeneration of the electrode. As redox indicator [Fe(CN_6_)^3−^]/[Fe(CN_6_)^4−^] was used for the measurement. The linear dynamic range from 10^3^ to 10^7^ CFU·mL^−1^ with a LOD of 10^3^ CFU·mL^−1^ were obtained by this setup.

### 3.3. Detection of Listeria monocytogenes by Electrochemical Biosensors

**Figure 2:**
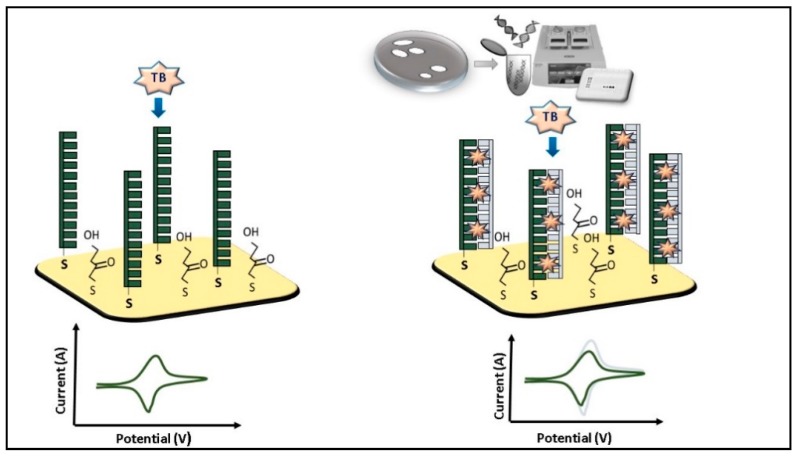
*Listeria monocytogenes* amplified DNA detection by electrochemical biosensor based on cycle voltammetry using as indicator Toluidine blue (TB) [39].

More studies on the detection of *L. monocytogenes* by electrochemical biosensors were published compared to those on detection of *Campylobacter*. DNA sensors have attracted the attention for the advantages of being cheap, rapid, and highly selective. Gao et al. [42] developed an electrochemical biosensor composed of a DNA probe that recognized the gene *actA*. The gene *actA* encodes for a *L. monocytogenes* surface protein actine assembly-inducing (ActA) that enables bacterial cells to propel through mammalian cells upon infection. The Au electrode was dipped in a mercaptoacetic acid solution to form self-assembled layer activated with a mix of N-(3-dimethylaminopropyl)-N′-ethylcarbodiimide hydrochloride (EDC) and N-hydroxysuccinimide (NHS). The DNA probe (bioreceptor) was immobilized on the electrode surface via amino links (Figure 2). Toluidine blue (TB) was used as an electrochemical indicator because it is able to discriminate ssDNA (DNA probe) from dsDNA as it intercalates double strands, but not single strands. The concentration range tested was from 1 × 10^−7^ to 8 × 10^−5^ mol·L^−1^. An increment of the anionic peak intensity proportional to the concentration of hybridized DNA target was observed.

In another study, Sun et al. [43], used methylene blue (MB), which covalently bind guanidine bases, as a hybridization indicator. The MB signal reduces after DNA hybridization because guanidine bases in dsDNA are not available. Voltammetric measurements were performed with the potassium ferricyanide K_3_[FE(CN)_6_] as a redox probe. The target was the gene *hly* that encodes a major virulence factor (sulfhydryl-activated pore-forming toxin) of *Listeria*. The biosensor was tested for the concentration range 1 × 10^−12^–1 × 10^−6^ mol·L^−1^. The detection limit was 2.9 × 10^−13^ mol·L^−1^.

Bifulco et al. [44] obtained a proportional decrease of the differential pulse voltammetry (DPV) signal with an increase in the concentration of the hybridized DNA. The DNA probe used was specific for the gene *inlA* which encodes for a cell wall protein, internalin A, in *L. monocytogenes.* The DNA probe was immobilized on the functionalized Au screen printed electrode via amino link. The detection was tested with both amplicons and whole DNA of *L. monocytogens* in a range of DNA concentrations from 25 ppm to 150 ppm. As expected, the electrochemical signal of MB, used as an electrochemical indicator, was inversely proportional to the DNA concentration. The detection discriminated between specific and non-specific binding. The time required for analysis was 60 min.

A recent study conducted by Yan et al. [45] developed a DNA probe to detect the gene *hly*, encoding the pore-forming cytolysin listeriolysin O, of *L. monocytogenes*. They tested a sensitive electrode composed of three-dimensional graphene, which showed increased conductivity, and an Au nanostructure, which improved the immobilization of the probe. The MB was used as electrochemical indicator in DPV measurements. The data showed a decrease of the current proportional to the increase of the hybridized DNA, when the sequence complementary to the probe and the amplicons were tested.

Kashihs et al. [46] reported a label-free electrochemical impedance biosensor for *L. monocytogens* detection. The specific probe, designed on *hly* gene, was covalently immobilized on poly-5carbocy indole (5C Pin) after surface activation by EDC and NHS. The change of the impedance was measured before and after hybridization. The linear dynamic range from 1 × 10^−12^ to 1 × 10^−4^ mol·L^−1^ and the detection limit of 2.34 × 10^−13^ mol·L^−1^ was observed.

An immuno-sensor for the detection of *L. monocytogenes* was constructed using screen-printed carbon electrodes modified with Au nanoparticles conjugated with an antibody specific for *L. monocytogenes* as a bioreceptor by Davis et al. [47]. *L. monocytogenes* was detected using a second antibody labelled with horse radish peroxydase enzyme (HRP). The immobilization of the antibody on Au nanoparticles generated an amperometric signal amplification. The sensitivity of the sensor was determined in blueberry samples spiked with *L. monocytogenes* in concentrations from 1 log to 5 log CFU·g^−1^.

In Table 1 are listed the LOD values obtained in the different electrochemical assays used to detect *Campylobacter* and *Listeria monocytogenes.*

## 4. Detection of *Campylobacter* spp. and *Listeria* by Optical Biosensors

### 4.1. Optical Biosensors

Optical biosensors provide an optical signal (color, chemiluminescence or fluorescence) that is generated directly by a bioreceptor and biomarker or through a recognition process. For instance, the formation of an antibody - antigen complex can be easily measured by the optical biosensor using an antibody labelled with a fluorescent probe. Alternatively, the recognition event, which does not generate directly an optical signal, may cause a change in the optical properties of the environment. In addition, some optical methods, like surface plasmon resonance (SPR) and surface-enhanced Raman spectroscopy, provide label-free detection of biological molecules.

The colorimetric optical signals may be observed by the naked eye. To provide quantitative measurements optical sensors integrate a photodetector (photodiodes, photomultipliers, CDD camera) that converts an optical signal to a measurable electrical signal. Main advantages of optical biosensors are that they are fast, sensitive, reliable, and easily adaptable to multiplex format. However, they are susceptibility to environmental interference that may cause photobleaching of photoactive molecules, and demand expensive filters and/or fragile optics.

### 4.2. Detection of Campylobacter by Optical Biosensors

**Figure 3:**
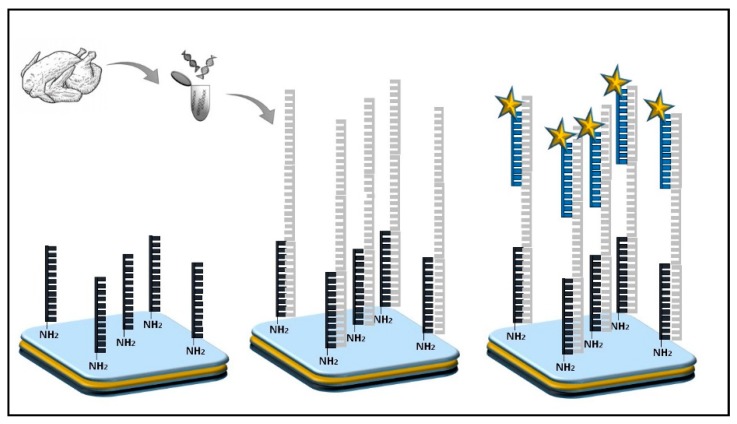
*Campylobatcer* spp. DNA detection by optical biosensor based on OLED [45]. Stars correspond to the fluorophore Alexa Fluor^®^ 430.

The majority of optical sensors for *Campylobacter* detection are genosensors that employ different DNA probes. Manzano et al. [48] used an organic light emission diode (OLED) to detect *Campylobacter* spp. in poultry meat samples. A specific DNA probe modified with an amino group at 5’ was used as a capture element and a DNA probe labelled with Alexa Fluor^®^ 430 (Thermo Fisher Scientific, Waltham, MA, USA) was used as a detection probe (Figure 3). The biosensor targeted a specific DNA sequence for the 16S rRNA gene of *Campylobacter* spp. The OLED produced a specific emission spectrum for the excitation of the Alexa Fluor^®^ 430 molecule upon target detection. The biosensor was validated with classical and molecular methods to confirm sensitivity and specificity. The system showed a good linear correlation in the tested range of concentrations with an R^2^ of 0.99 and a sensitivity of 0.37 ng·µL^−1^ DNA indicating a suitable utilization for real sample analysis. In addition, no cross-reactivity was observed using genomic DNA extracted from non-related microorganisms.

Shams et al. [49] developed gold nanorods functionalized with a DNA probe (GNRs-DNA) specific for the detection of *cadF* gene which encodes a cell membrane protein CadF in *C. jejuni* and *C. coli*. They measured the variation between the SPR before and after the hybridization with specific amplicons of 95 bp obtained by PCR used as a positive target. The SPR bands obtained using a negative control did not show any change, demonstrating good specificity of the test. The method applied directly to stool samples showed a LOD of 10^2^ copy number·mL^−1^. A similar detection limit was obtained by PCR (10^3^ copy number·mL^−1^) and qPCR (10^2^ copy number·mL^−1^).

Gnanaprakasa et al. [50] developed a platform for the detection of *C. jejuni* using DNA probes specific for the *hipO* (hippuricase) gene. Two different optical methods were used for the quantification of the target, the SPR and the diffraction optical technology (DOT). DNA probes were thiolated for the application on SPR gold chips and biotinylated for the application on DOT. MCH was employed as a spacer to displace DNA probes and single strand DNA not bound to the gold surface, and to reduce not specific signals. The SPR showed advantages over the DOT method since provided a lower LOD of 2.5 × 10^−9^ mol·L^−1^ comparing to 5 × 10^−9^ mol·L^−1^ obtained with DOT. In addition, SPR assay offered the possibility of reutilization of the sensor chip because the method did not damage DNA probes.

Wei et al. [51] used antibodies for the detection of whole cells of *C. jejuni* with the optical biosensors. The gold surface of a SPR chip was functionalized with an antibody specific for *C. jejuni* cells. Wash water obtained from broiler samples were tested. The detection limit of 10^3^ CFU·mL^−1^ was observed. Targeting the whole cell instead of amplicons, the detection of *Campylobacter* was enabled without biomarker (DNA) extraction and purification, which simplified the protocol.

Several strategies have been tested to improve the sensitivity of optical biosensors. Masdor et al. [52] investigated a SPR technique for the detection of *C. jejuni* in food samples using a specific polyclonal antibody as a recognition element. After the activation of the functionalized gold surface with a NHS/EDC solution, the antibody was immobilized on parallel spots at different concentrations. Before the addition of bacterial cells, albumin was used as a blocking agent. The detection was performed using the recognition antibody alone or conjugated to Au nanoparticles to improve the LOD, using a single concentration of *C. jejuni* cells (1 × 10^7^ CFU·mL^−1^). The sandwich assay which used antibodies without AuNPs showed a LOD of 4 × 10^4^ CFU·mL^−1^, while the sandwich assay with antibodies conjugated to AuNPs unexpectedly showed a LOD of 8 × 10^5^ CFU·mL^−1^, one log higher than obtained with antibodies alone. Authors showed that the utilization of antibodies conjugated with Au NPs failed to improve the signal in this platform.

Kim et al. [53] used gold nanoparticles functionalized with aptamers, that specifically bind whole cells of *C. jejuni* and *C. coli,* to develop a sensitive colorimetric method. Before the test, aptamers were adsorbed on gold nanoparticles. The solution was of red color because adsorbed aptamers prevented nanoparticle aggregation. In the presence of the *Campylobacter* cells, the aptamer molecules desorbed from the NPs to bind bacteria, and as a consequence, the AuNPs aggregated and the color of solution turned to purple. The color changes were quantified by the UV-Vis spectrophotometer, showing that the changes in adsorption spectrum were proportional to the concentration of the target bacteria. The assay was tested on contaminated chicken samples and showed LODs of 5.6 × 10^5^ CFU·mL^−1^ and 7.2 × 10^5^ CFU·mL^−1^ for *C. coli* and *C. jejuni,* respectively.

### 4.3. Detection of Listeria monocytogenes by Optical Biosensors

**Figure 4:**
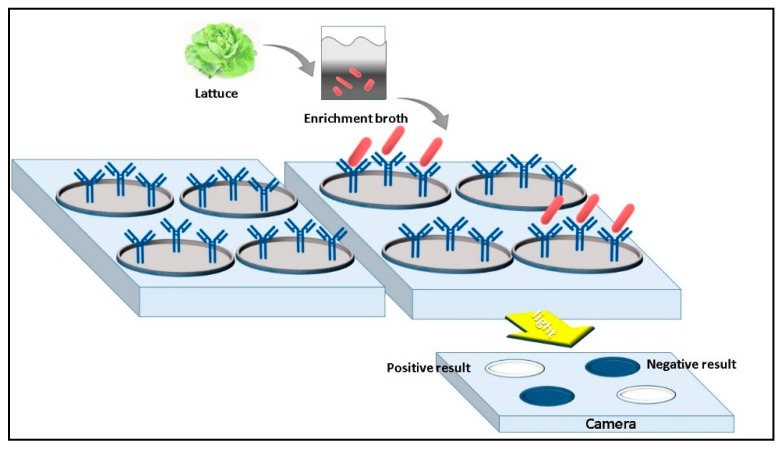
*Listeria monocytogenes* cells detection by SPR biosensor based on Antibody bioreceptor [51].

Morlay et al. [54] developed a label-free system based on SPR imaging (SPRi) coupled with an immunosensor specific for *L. monocytogenes* detection. A biochip with a gold surface was functionalized with seven different polyclonal antibodies. BSA solution was used as a blocking agent to avoid nonspecific binding. During the analysis, the SPRi signal of the biochip was monitored in real time during the injection of various bacterial concentrations. The antibody successfully bound bacterial cells in both pure culture and lettuce samples inoculated with three *L. monocytogenes* strains (from 17 to 25 CFU·g^−1^) (Figure 4). No cross-reactivity was observed using non-related bacteria. The proposed method permitted the detection of *L. monocytogenes* in 30 min but demanded 24 h enrichment step prior to analysis.

Liu et al. [55] used an aptamer as a recognition element for the specific binding of *L. monocytogenes* and an antibody labelled whit MnO_2_ that catalyses the oxidation of TMB for the detection. An AuNP solution was added to react with the TMB^2+^ leading to a color change. The reaction was followed by a UV-Vis spectrophotometer for the evaluation of the Localized Surface Plasmon Resonance (LSPR) parameter. The results showed that peak shifts were correlated to the *L. monocytogenes* concentration. Moreover, semi-qualitative analysis obtained by the naked eye detected bacteria with the low limit of detection of 10 CFU·mL^−1^. Pork meat samples were used to validate the detection system. Positive samples were obtained by inoculating *L. monocytogenes* at various concentrations ranging from 5 × 10 to 5 × 10^5^ CFU·mL^−1^ to meat samples. Meat without inoculum served as negative samples. *L. monocytogenes* was detected at a low concentration of 50 CFU·mL^−1^, showing the feasibility of the system to analyze real samples.

Ohk et al. [56] optimized the coating of fiber optics with streptavidin to attach antibodies and to build a biosensor for the detection of pathogens. In 2013 Ohk et al. [57] realized an optical fiber functionalized with a biotinylated capture antibody linked to the streptavidin coating via biotin-streptavidin interaction. Monoclonal antibodies directed against *E. coli*, *Salmonella* and *L. monocytogenes* were labeled with Alexa Fluor^®^ 647 used to produce a fluorescent signal. Meat samples were artificially spiked with pathogens separately or in a mixture (*E. coli*, *Salmonella* and *L. monocytogenes*) to obtain a final concentration of 10^2^ CFU·mL^−1^ in single or mixed culture. After 2 h incubation, samples were analyzed with the fiber optic biosensor which used a sandwich format. The LOD of the system was 10^3^ CFU·mL^−1^.

Colorimetric immune-assays to detect *L. monocytogenes* by the naked eye are not widely reported mainly because of their low sensitivity that is not suitable for the detection of *Listeria* in food where the concentration is low. To improve this method Zhang et al. [58] proposed to couple Fe_3_O_4_ NPs (which have a peroxidase like activity) and antibiotic vancomycin, which recognizes and binds the D-Alanyl-D-Alanine motive of cell wall of *L. monocytogenes*. An aptamer specific for the internalin A protein, of the cell wall of *L. monocytogenes*, was conjugated with Fe_3_O_4_ nanoparticle clusters to amplify the signal. The formation of a sandwich in which bacterial cells were contemporary bounded to vancomycin conjugated with BSA, and the aptamer conjugated to Fe_3_O_4_ nanoparticle cluster (which catalyzes the production of color) allowed for detection by the naked eye. The biosensor was tested on milk samples spiked with known amounts of the pathogen. The detection of *L. monocytogenes* was possible in a linear range from 5.4 × 10^3^–5.4 × 10^8^ CFU·mL^−1^ and with a visual LOD of 5.4 × 10^3^ CFU·mL^−1^.

In Table 2 are reported the LOD obtained with the optical biosensors proposed for the detection of *Campylobacter* and *Listeria monocytogenes.*

## 5. Conclusions

Official methods for detection of *Campylobacter* spp. and *L. monocytogenes* are sensitive and accurate but also time-consuming (up to one week) and thus provide only limited practical applications [59,60]. In last decades, many efforts have been devoted to the biosensor devices for detection of these two pathogens. Biosensors offer high sensitivity, selectivity and rapidity, and also low cost, real time measurements and non-destructive sensing. To elaborate optical and electrochemical sensors various recognition elements were employed including DNA probe, antibodies, aptamers, and antibiotics. Because both *Campylobacter* spp. and *L. monocytogenes* may cause infectious diseases even at low titer it is important to note that some biosensors provide LOD as low as 1 CFU·mL^−1^. However, to reach market the biosensors for detection of *Campylobacter* spp. and *L. monocytogenes* still have to be optimized. Sensors based on aptamers and DNA sequences are generally the most sensitive compared to antibody- based biosensors. Moreover, other challenges for the biosensors are to work under simultaneous multiple conditions, and to be more robust for long-term use. The goal would be the construction of a reusable biosensor to reduce the cost of analyses.

The future sensing technology challenges will be the miniaturization of the device components and their standardization for the most effective application in preventing and surveillance programs of foodborne pathogens.

To reach the goal of sensitive, rapid, cheap and specific biosensors some points have to be optimized although advances have been made in this field. Nowadays, DNA based biosensors have obtained good results in terms of stability, reproducibility, sensitivity and availability at market level.

The rapid progress and multitude of novel electrochemical and optical assays together with increased demand guarantee the promising future of biosensors for food security.

## Figures and Tables

**Table 1:** Electrochemical biosensor for the detection of *Campylobacter* and *Listeria monocytogenes.*

	Bioreceptor	Biomarker	Method	LOD	Matrix	References
*Campylobacter* spp.	Antibody	Cell	Amperometry	1 cell	Bacterial suspension	[33]
	Antibody	Cell	Potentiometry	2.1 × 10^4^ CFU·mL^−1^	poultry washing water	[34]
	Antibody	Cell	Amperometry	400 cell·mL^−1^	milk sample	[35]
	DNA probe	Amplicon	Amperometry	9 × 10^−11^ mol·L^−1^	raw poultry meat	[36]
	Antibody	Cell	Impedimetry	10^3^ CFU·mL^−1^	stools	[41]
*Listeria monocytogenes*	DNA probe	*actA* gene	Amperometry	not evaluated	DNA	[42]
	DNA probe	*hly* gene	Amperometry	2.9 × 10^−13^ mol·L^−1^	fish	[43]
	DNA probe	*inlA* gene	Differential pulse Voltammetry	not evaluated	DNA	[44]
	DNA probe	*hly* gene	Differential pulse Voltammetry	3.3 × 10^−15^ mol·L^−1^	DNA	[45]
	DNA probe	*hlyA* gene	Impedimetry	10^−13^ mol·L^−1^	DNA	[46]
	Antibody	Cell	Voltammetry	2 log CFU·mL^−1^	blueberry	[47]

**Table 2:** Optical detection for *Campylobacter* and *Listeria monocytogenes*.

	Bioreceptor	Biomarker	Method	LOD	Matrix	References
*Campylobacter* spp.	DNA probe	DNA	OLED	0.37 ng·µL^−1^	poultry meat	[48]
	DNA probe	DNA	SPR	10^2^ copy·mL^−1^	DNA	[49]
	DNA probe	DNA	SPR	2.5 × 10^−9^ mol·L^−1^	DNA	[50]
	Antibody	cells	SPR	10^3^ CFU·mL^−1^	washing water	[51]
	Antibody	cells	SPR	4 × 10^4^ CFU·mL^−1^	bacterial suspension	[52]
	Aptamer	cells	Colorimetric aptasensor	7.2 × 10^5^ CFU·mL^−1^ (*C. jejuni*) 5.6 × 10^5^ CFU·mL^−1^ (*C. coli*)	chicken carcass	[53]
*Listeria monocytogenes*	Antibody	cells	SPR	not evaluated	spiked lettuce	[54]
	Aptamer	cells	Colorimetric assay	10 CFU·mL^−1^	spiked pork	[55]
	Antibody	cells	Optical fiber	10^3^ CFU·mL^−1^	chicken and turkey	[56]
	Aptamer	cells	Optical fiber	5.4 × 10^3^ CFU·mL^−1^	milk	[57]
